# Proportion of Community-Dwelling Individuals Older Than 70 Years Eligible for Lecanemab Initiation: The Gothenburg H70 Birth Cohort Study

**DOI:** 10.1212/WNL.0000000000209402

**Published:** 2024-04-09

**Authors:** Anna Dittrich, Eric Westman, Sara Shams, Tobias Skillbäck, Henrik Zetterberg, Kaj Blennow, Anna Zettergren, Ingmar Skoog, Silke Kern

**Affiliations:** From the Neuropsychiatric Epidemiology Unit (A.D., T.S., A.Z., I.S., S.K.), Sahlgrenska Academy, University of Gothenburg; Department of Psychiatry Cognition and Old Age Psychiatry (A.D., I.S., S.K.), Sahlgrenska University Hospital, Mölndal; Division of Clinical Geriatrics (E.W.), Department of Neurobiology, Care Sciences and Society (NVS), Karolinska University Hospital, Stockholm, Sweden; Department of Neuroimaging (E.W.), Centre for Neuroimaging Sciences, Institute of Psychiatry, Psychology and Neuroscience, King's College London, United Kingdom; Care Sciences and Society (S.S.), Karolinska Institutet, and Department of Radiology; Department of Clinical Neuroscience (S.S.), Karolinska University Hospital, Stockholm Sweden; Department of Radiology (S.S.), Stanford University, CA; Department of Psychiatry and Neurochemistry (H.Z., K.B.), Institute of Neuroscience and Physiology, the Sahlgrenska Academy at the University of Gothenburg; Clinical Neurochemistry Laboratory (H.Z., K.B.), Sahlgrenska University Hospital, Mölndal Sweden; UK Dementia Research Institute at UCL (H.Z.); Department of Neurodegenerative Disease (H.Z.), UCL Institute of Neurology, London United Kingdom; Hong Kong Center for Neurodegenerative Diseases (H.Z.), Hong Kong, China; Wisconsin Alzheimer's Disease Research Center (H.Z.), University of Wisconsin School of Medicine and Public Health, University of Wisconsin-Madison; Paris Brain Institute (K.B.), ICM, Pitié-Salpêtrière Hospital, Sorbonne University, Paris, France; and Neurodegenerative Disorder Research Center (K.B.), Division of Life Sciences and Medicine, and Department of Neurology, Institute on Aging and Brain Disorders, University of Science and Technology of China and First Affiliated Hospital of USTC, Hefei, P.R. China.

## Abstract

**Objectives:**

To determine the prevalence of individuals with Alzheimer disease (AD) eligible for treatment with the recently FDA-approved lecanemab based on data from a population-based sample of 70-year-olds and extrapolate an estimation of individuals eligible in Europe and the United States.

**Methods:**

Participants from the Gothenburg H70 Birth Cohort Study with clinical data, CSF-amyloid beta 42, and brain MRI analysis were evaluated for eligibility to receive lecanemab treatment according to FDA-approved recommendations, noting factors requiring special consideration. Results were used to extrapolate the number of eligible individuals in Europe and the United States using public demographic data.

**Results:**

Thirty (10.3%) of 290 participants met the indication for treatment of whom 18 (6.2%) were eligible and did not present factors requiring special consideration. Our estimate that 6.2% of all 70-year-olds in the full cohort are eligible for treatment extrapolates to an approximation that around 5.9 million Europeans and 2.2 million US residents could be eligible.

**Discussion:**

Information on proportion of individuals eligible for AD treatment with lecanemab in the general public is limited. We provide information on 70-year-olds in Sweden and extrapolate these data to Europe and the United States. This study opens for larger studies on this proportion and implementation of lecanemab treatment.

## Introduction

Alzheimer dementia (AD) is the leading cause of dementia, proposedly caused by the cerebral deposition of amyloid beta (Aβ)-plaques leading to neurodegeneration.^1^ Lecanemab is an antibody directed against aggregated forms of Aβ, approved by The Food and Drug Administration (FDA) for the treatment of AD.^2^

The treatment has been associated with increased risk of amyloid-related imaging abnormalities (ARIA) on MRI and serious intracerebral hemorrhage.^3^ Therefore, factors associated with increased risk should be considered when using lecanemab.^2^ These factors are detailed as findings on brain MRI indicating an increased risk of intracerebral hemorrhage, concurrent antithrombotic medication, and *APOE ε4* homozygosity. In addition, in the clinical trials leading to approval, patients with more severe vascular lesions on MRI were excluded. Information on the proportion of individuals who are eligible for treatment with lecanemab, according to FDAs criteria in the general public is limited. The objective of this study was to examine eligibility in a population-based sample of 70-year-olds and extrapolate our results to estimate the magnitude of individuals eligible in the United States and EU, based on public data and the FDA-approved prescribing information for lecanemab (LEQEMBI).^2^

## Methods

We determined the proportion of individuals meeting indication for lecanemab in a population-based cohort of the Gothenburg H70 Birth Cohort Study (H70-BCS), born 1944. First, identifying individuals meeting indication, and second identifying individuals presenting factors requiring special consideration, which may exclude them from treatment (factors presented in Table 1).

**Table 1:** Prevalence of Factors Which May Require Consideration in Lecanemab Treatment Among the 30 Eligible Participants From the Population-Based Gothenburg H70 Birth Cohort Study of 1944

	n (%)
Stroke in medical history	2 (7)
Any anticoagulant medication	2 (7)
Homozygote for *APOEε4*	4 (13)
Superficial siderosis	0 (0)
Microbleeds >4	1 (3)
Lacunes	5 (17)
Fazekas score = 3	4 (13)
Signs of intracerebral hemorrhage	1 (3)

LEQEMBI is indicated for treatment of AD with mild cognitive impairment (MCI) or mild dementia, without any further definition. Here, we define MCI and mild dementia as a clinical dementia rating (CDR) of 0.5–1. AD was defined as amyloid positivity, through CSF-Aβ42 < 530 pg/mL, based on a previous study.^4^

All citizens of Gothenburg born on specific birth dates in 1944 were invited, with no exclusion criteria.^5^ Nurses with psychiatric training examined participants, including medical background, blood sampling, and cognitive testing. All participants without contraindications were invited to undergo a brain MRI and lumbar puncture (LP).^5,6^ Anticoagulants were defined as any medication with an ATC code under B01. Participants were genotyped for *APOE ε4*, and Aβ42 concentration in CSF was measured using INNOTEST Aβ1-42.^7,8^

### Standard Protocol Approvals, Registrations, and Patient Consents

This study was conducted according to the Helsinki Declaration approved by the Regional Ethical Review Board in Gothenburg. All the participants and/or their close relatives gave written consent before any study-related procedures were performed.

### Data Availability

Anonymized data can be obtained by reasonable request from any qualified investigator.

## Results

### H70 Birth Cohort Proportion on Individuals Who Are Eligible for Lecanemab Treatment

Twelve-hundred three (response rate 72.2%) accepted participation of whom 290 had complete data on relevant variables (Figure).^5^ Thirty of the 290 were amyloid-positive and had MCI or mild dementia. Twelve of the 30 eligible participants presented 1 or several items requiring special consideration before treatment (Table 1). Taken together, in the H70-BCS, 18 of the 290 study participants (6.2%) were eligible for lecanemab treatment without any factor indicating risk of adverse events (Figure).

**Figure:**
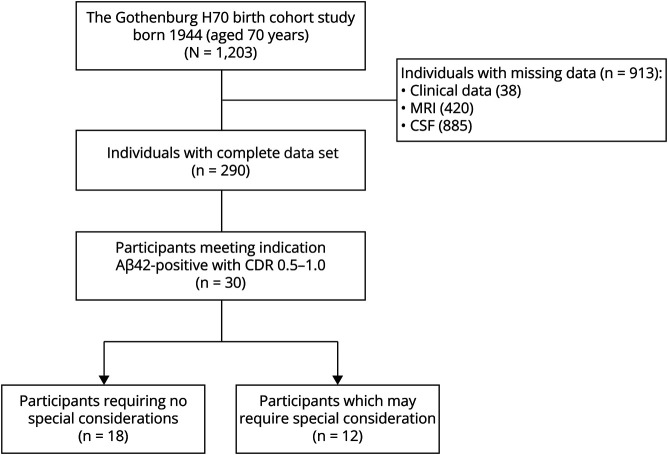
Study Flowchart Detailing How Many Participants of the Gothenburg H70 Birth Cohort Study of 1944 Who Had Complete Data Sets on Clinical, MRI, and CSF Variables; How Many Who Met the Indication for Lecanemab Treatment; and How Many Who Did Not Present Any Factor Which Required Special Consideration for the Treatment

Clinical characteristics of the sample (n = 290) were compared with the 913 individuals from the full birth cohort to assess representativity of the sample (Table 2). The sample had a small overrepresentation of men (52% vs 45%). Owing to a contraindication of some anticoagulant medications to perform LP, individuals with anticoagulant treatment or a medical history of cerebral stroke were overrepresented among those with missing data. No difference was seen on other characteristics, including CDR or *APOE ε4* homozygosity.

**Table 2:** Clinical Characteristics of Individuals in the Gothenburg H70 Birth Cohort Study of 1944 (n = 1203) With Complete Data Sets or Missing Data on Any Variable

	Missing data on any variable (n = 913)	No missing data on any variable (n = 290)
Male sex, n (%)	409 (45)	150 (52)^a^
Education, years in school	13 (10–16)	12 (10–15)
Household monthly income, SEK	34000 (24000–50000)	33200 (22000–50000)
CDR 0.5–1, n (%)	176 (20)	60 (21)
MMSE score	29 (29–30)	29 (28–30)
10-word immediate recall test	5 (4–7)	5 (4–7)
Body mass index, kg/m^2^	25.5 (23.1–28.8)	24.9 (22.9–27.9)
Systolic blood pressure, mm Hg	140 (126–150)	140 (125–150)
Diastolic blood pressure, mm Hg	80 (70–85)	80 (70–85)
Dementia diagnose, n (%)	26 (3)	4 (1)
Previous cerebral stroke, n (%)	69 (8)	8 (3)^a^
Hypertension, n (%)	633 (70)	210 (72)
Diabetes, n (%)	152 (17)	35 (12)
Any anticoagulant medication, n (%)	229 (25)	46 (16)^a^
*APOEε4* heterozygosity, n (%)	240 (27)	97 (33)
*APOEε4* homozygosity, n (%)	30 (3)	11 (4)

Categorical variables presented as n (%) and continuous variables as median (IQR).

Statistical tests used were χ^2^, Fisher exact test, and Mann-Whitney *U* test.

Missing data on Household monthly income (n = 296), CDR (n = 11), MMSE (n = 25), 10-word immediate recall test (n = 37), body mass index (n = 36), blood pressure (n = 7), any anticoagulant medication (n = 3), and *APOEε4* homozygosity (n = 38).

a Statistical difference between groups with *p* < 0.05.

### Estimating Proportion in Similar Populations Through Extrapolation and Public Data

Based on the proportion of individuals in the H70-BCS eligible for lecanemab treatment, without factors requiring special consideration, an estimate of magnitude was extrapolated in Europe and the United States based on the following assumptions (details in eAppendix 1): (1) The sample with complete data in this study is representative of the full H70-BCS. (2) Participants in the H70-BCS are representative of 70-year-olds in Sweden. (3) Swedish residents are equally affected by AD as other residents in Europe and the United States.^9^ (4) Proportion of individuals who are eligible is similar in 70-year-olds and older individuals because factors requiring special considerations regarding risk of adverse events increase with age to a similar magnitude as the prevalence of AD. This can be motivated because prevalence of factors such as stroke and anticoagulant treatment due to atrial fibrillation increase drastically with age.^10^

According to public data from “Our world in data,” EU currently has 95 million citizens between 70 and 89 years and the United States has 35 million.^11^ Based on this, the total number of individuals eligible for lecanemab treatment in the EU and the United States would account for approximately 5.9 million and 2.2 million individuals, respectively.

## Discussion

We determined the proportion of 70-year-olds eligible for lecanemab treatment in the H70-BCS through examinations, including cognitive function, medical history, genotyping, CSF analysis, and brain imaging, and found that 6.2% met the indication without any factor requiring special consideration.^2^ Assuming a similar proportion, and a similar ratio of individuals with AD without complicating factors in other parts of Europe and the United States, this could indicate that approximately 5.9 million Europeans and 2.2 million US residents may be eligible for treatment.

Studies on the absolute proportion of individuals in Europe and the United States who are eligible for treatment are few. A population-based study from the United States recently found that a limited proportion of older adults met the strict clinical trial criteria for lecanemab treatment.^12^ Our assessment of eligibility using the FDA-approved prescribing information resulted in higher proportion eligible for treatment. On a larger scale, it was recently estimated that 5.4 million Europeans are eligible for lecanemab treatment, based on public data, and an assumption that one-third of individuals with AD and MCI would qualify for treatment.^13^ Our estimate of 5.9 million Europeans aligns well with this, although extrapolated from a population-based sample with individual clinical and MRI data. Together, these independent estimations support each other and indicate that our extrapolation holds some validity on the larger scale. Furthermore, our extrapolation suggests that 2.2 of the 6.7 million Americans with Alzheimer dementia may be candidates for treatment.^14^

The presented estimates of eligibility in EU and the United States are crude and should not be used for future policy making or direction of funds for future care. However, our study contributes to data based on the current FDA-approved prescribing information. Furthermore, we analyzed Aβ42 in CSF, which can be influenced by other diseases in the CNS, and may differ slightly from using PET.^15^ As our extrapolations are based on 70-year-olds, this may result in an underestimation of eligibility. Conversely, the proportion of individuals with stroke and anticoagulant treatment was lower than in the full H70-BCS, which could lead to a slight overestimation of eligibility. Thus, these 2 concerns could mitigate each other. Our sample with full data was comparatively small (290 of 1,203) and not ethnically diverse, and risk factors for AD may also differ from country to country and influence generalizability. However, there were no other differences between participants with complete or missing data, and previous estimates on the proportion eligible in Europe have presented similar numbers through other methods.

In conclusion, we found that 6.2% of the 70-year-olds in the H70 Birth Cohort Study born 1944 are eligible for lecanemab treatment. Other large population-based studies should determine the proportion in other settings to provide further information regarding the magnitude of individuals who may benefit from lecanemab treatment.

## Appendix Authors

**Table:**

Name	Location	Contribution
Anna Dittrich, MSc	Neuropsychiatric Epidemiology Unit, Sahlgrenska Academy, University of Gothenburg; Department of Psychiatry Cognition and Old Age Psychiatry, Sahlgrenska University Hospital, Mölndal Sweden	Drafting/revision of the manuscript for content, including medical writing for content; study concept or design; analysis or interpretation of data
Eric Westman, PhD	Division of Clinical Geriatrics, Department of Neurobiology, Care Sciences and Society (NVS), Karolinska University Hospital, Stockholm Sweden; Department of Neuroimaging, Centre for Neuroimaging Sciences, Institute of Psychiatry, Psychology and Neuroscience, King's College London, United Kingdom	Drafting/revision of the manuscript for content, including medical writing for content; major role in the acquisition of data; study concept or design; analysis or interpretation of data
Sara Shams, MD, PhD	Care Sciences and Society, Karolinska Institutet, and Department of Radiology; Department of Clinical Neuroscience, Karolinska University Hospital, Stockholm Sweden; Department of Radiology, Stanford University, CA	Drafting/revision of the manuscript for content, including medical writing for content; major role in the acquisition of data; analysis or interpretation of data
Tobias Skillbäck, MD, PhD	Neuropsychiatric Epidemiology Unit, Sahlgrenska Academy, University of Gothenburg, Gothenburg Sweden	Drafting/revision of the manuscript for content, including medical writing for content; analysis or interpretation of data
Henrik Zetterberg, MD, PhD	Department of Psychiatry and Neurochemistry, Institute of Neuroscience and Physiology, the Sahlgrenska Academy at the University of Gothenburg, Mölndal Campus; Clinical Neurochemistry Laboratory, Sahlgrenska University Hospital, Mölndal Sweden; UK Dementia Research Institute at UCL; Department of Neurodegenerative Disease, UCL Institute of Neurology, London, United Kingdom; Hong Kong Center for Neurodegenerative Diseases, Hong Kong, China; Wisconsin Alzheimer's Disease Research Center, University of Wisconsin School of Medicine and Public Health, University of Wisconsin-Madison	Drafting/revision of the manuscript for content, including medical writing for content; major role in the acquisition of data; analysis or interpretation of data
Kaj Blennow, MD, PhD	Department of Psychiatry and Neurochemistry, Institute of Neuroscience and Physiology, the Sahlgrenska Academy at the University of Gothenburg, Mölndal Campus; Clinical Neurochemistry Laboratory, Sahlgrenska University Hospital, Mölndal Sweden; Paris Brain Institute, ICM, Pitié-Salpêtrière Hospital, Sorbonne University, France; Neurodegenerative Disorder Research Center, Division of Life Sciences and Medicine, and Department of Neurology, Institute on Aging and Brain Disorders, University of Science and Technology of China and First Affiliated Hospital of USTC, Hefei, P.R. China	Drafting/revision of the manuscript for content, including medical writing for content; major role in the acquisition of data; analysis or interpretation of data
Anna Zettergren, PhD	Neuropsychiatric Epidemiology Unit, Sahlgrenska Academy, University of Gothenburg, Sweden	Drafting/revision of the manuscript for content, including medical writing for content; major role in the acquisition of data; analysis or interpretation of data
Ingmar Skoog, MD, PhD	Neuropsychiatric Epidemiology Unit, Sahlgrenska Academy, University of Gothenburg; Department of Psychiatry Cognition and Old Age Psychiatry, Sahlgrenska University Hospital, Mölndal Sweden	Drafting/revision of the manuscript for content, including medical writing for content; major role in the acquisition of data; study concept or design; analysis or interpretation of data
Silke Kern, MD, PhD	Neuropsychiatric Epidemiology Unit, Sahlgrenska Academy, University of Gothenburg; Department of Psychiatry Cognition and Old Age Psychiatry, Sahlgrenska University Hospital, Mölndal Sweden	Drafting/revision of the manuscript for content, including medical writing for content; major role in the acquisition of data; study concept or design; analysis or interpretation of data
